# Faster chemical mapping assisted by computer vision: insights from glass and ice core samples†

**DOI:** 10.1039/d5an00325c

**Published:** 2025-06-19

**Authors:** Piers Larkman, Sebastiano Vascon, Martin Šala, Nicolas Stoll, Carlo Barbante, Pascal Bohleber

**Affiliations:** a Department of Environmental Sciences, Informatics and Statistics, Ca'Foscari University of Venice Venice Italy piersmichael.larkman@unive.it; b Department of Geosciences, Alfred Wegener Institute Helmholtz Centre for Polar and Marine Research Bremerhaven Germany; c Department of Analytical Chemistry, National Institute of Chemistry Ljubljana Slovenia; d Department of Earth and Space Science, University of Washington Seattle USA; e Institute of Polar Sciences, National Research Council (CNR-ISP) Venice Mestre Italy; f Goethe University Frankfurt am Main Frankfurt am Main Germany

## Abstract

Recent advances in high-repetition-rate lasers and fast aerosol transfer facilitate laser ablation inductively coupled plasma mass spectrometry (LA-ICP-MS) mapping rates of up to megapixels per hour, however, practical limits in time and resources still hamper mapping the chemistry of square centimetre or larger areas of target samples at high resolutions. This is especially relevant for the analysis of deep sections of polar ice cores, motivating exploration of approaches to improve the efficiency of LA-ICP-MS data collection for large-area mapping. Assisted by computer vision, and demonstrated on glass and ice samples, we show how an informed experimental design coupled with computational post processing can contribute to large reductions in measurement times and lead to associated increases in measurement areas. Using various inpainting techniques, we demonstrate how the collection of data can be reduced by up to two thirds while still capturing spatial variability. Although motivated by ice core analysis, these approaches are generalisable to other target matrices and represent a new approach to large-area LA-ICP-MS mapping.

## Abstract 2

Saving time and resource in measurement – image repairing neural networks can be applied to speed up two-dimensional chemical mapping of samples.

## Graphical abstract

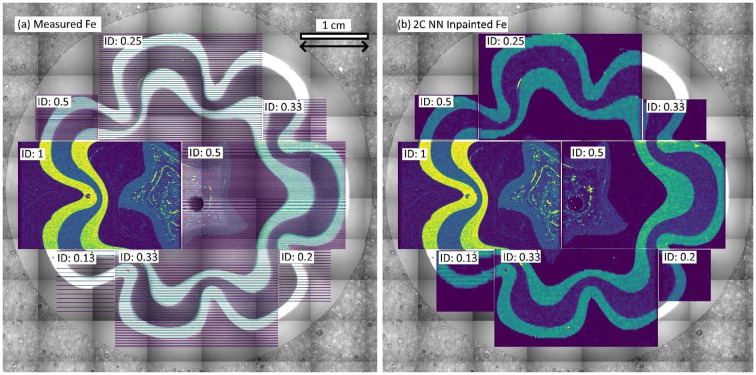

## 1 Introduction

Laser ablation inductively coupled plasma mass spectrometry (LA-ICP-MS) has become a key technology for investigating the spatial distribution of elemental and isotopic chemistry for various environmental, biological, and manufactured samples.^1,2^ Ongoing advancements to the technique have focused on increasing analysis speed to facilitate high-resolution acquisition, high mapping rates, and reducing cost.^3^ Development of high repetition rate laser systems, fast aerosol transport, and fast ion detection have contributed to increased mapping rates and image quality.^4,5^ The use of high-frequency lasers facilitates a significant decrease in measurement times^5^ and pairs well with time of flight-ICP-MS systems to avoid the restriction of mass cycling imposed by scanning mass spectrometers.^6^ However, practical constraints, such as ensuring reasonable measurement times and resource consumption, still hamper the mapping of large areas of square centimetres or larger. To further increase the speed and efficiency at which LA-ICP-MS data can be collected, improving the state-of-the-art is vital.

Applied to approximate missing data in several fields, inpainting approaches that fill in damaged or missing parts of an image,^7,8^ represent a potential approach to facilitate large area measurements using LA-ICP-MS. There are many inpainting techniques, with both traditional^9^ and deep learning^10^ approaches implemented and refined. Viewing LA-ICP-MS spectral data as an elemental or chemical image motivates experiments that collect sparse data using LA-ICP-MS and subsequent approximation of missing regions using inpainting techniques. Such experimental design removes the requirement to fully measure an area, thus facilitating faster mapping of larger areas, but is yet to be tested.

A practical application that can benefit from increased measurement efficiency using such approaches is the mapping of ice core samples using LA-ICP-MS. Current ice core drilling efforts target the acquisition of old ice samples, of approximately 1.5 million years old, from the bottom of the Antarctic ice sheet.^11^ The deepest samples, amounting to hundreds of metres of ice with more than 10 000 years of climate history contained per vertical metre,^12^ will require high-resolution spatial analysis for thorough interpretation of climate information.^13^ Application of LA-ICP-MS to measure elemental impurities in polar ice core samples has returned spatial information at to-date unmatched micrometre resolutions.^13–15^ Such resolutions will be crucial for collecting and interpreting the wealth of paleoclimate data^16^ contained in deep ice samples, particularly as 2D mapping, over collection of 1D parallel lines along the down-core axis, is vital for properly interpreting preserved signals.^17–20^ Therefore, LA-ICP-MS measurements on ice must be routinely sped up to gather and manage the large volume of high-resolution data needed to interpret climate signals from deep ice samples best.

The utility of computer vision techniques for analysis of chemical and optical data collected on ice core samples has been previously demonstrated.^21,22^ Interpolation of chemical LA-ICP-MS data has been carried out to fill in gaps in data collected at resolutions of hundreds-of-microns^23^ and the ice grain boundary (GB) network has been automatically masked from optical data.^22^ To demonstrate a further application of computer vision techniques to ice core data, and to aid LA-ICP-MS analysis, modern inpainting techniques can be applied as a postprocessing step. Given that GBs are visible in optical images and the dominant localisation of soluble chemistry at GBs, optical data can be a further useful reference for such analysis.

Here we demonstrate how carefully designed experimental LA-ICP-MS data collection, collecting sparse spectral data instead of full maps, can reduce measurement times, without compromising the extent of the surface area mapped, or the mapping resolution. Data is deliberately under-sampled during measurement and both traditional and machine learning inpainting approaches are applied to approximate the missing data. We utilise a murrina glass sample (Murano, Italy), which are often used to demonstrate developments to LA-ICP-MS measurement procedures due to regions showing well-understood variation in elemental concentrations,^24,25^ as a proof of concept application. Building on this demonstrative case, this novel approach of sparse measurement and subsequent inpainting is applied to ice core samples to show an example of practical use. We first validate the performance of the inpainting approaches before presenting data collected and processed using this novel approach. The utility of optical data, which can be rapidly collected and often shows variability which correlates with regions of variable chemistry, to guide inpainting reconstructions is also demonstrated.

## 2 Dataset collection and processing

### 2.1 Overview

**Fig. 1:**
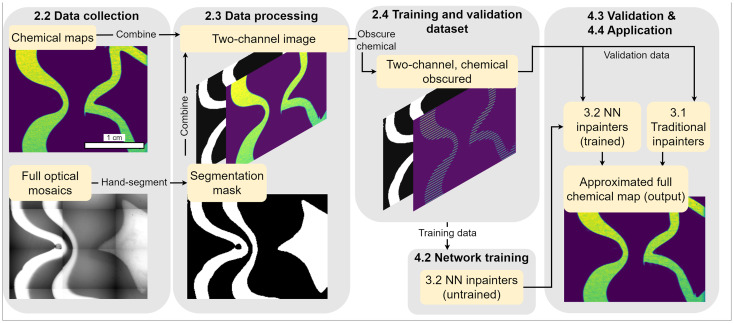
Diagram illustrating data collection and usage, using murrina glass data as an example. Optical and spectral data are collected using an LA-ICP-MS system, and the optical data have distinct regions segmented. For training and validating the inpainting approaches, full chemical maps are collected and processed into training and validation datasets by removing certain rows from chemical maps to produce sparse data as a target for inpainting. Some masked chemical and optical data are isolated for assessing the performance of the four inpainting approaches. The rest of the data are used as training data for the two inpainting approaches which utilise NNs. The trained NNs and two traditional approaches make up a set of four possible inpainting approaches which are used to approximate full chemical data. Application data are experimentally collected as sparse chemical maps, and are input directly to the inpainters.

We demonstrate the utility of inpainting methods to reduce demands on time and consumables for large area LA-ICP-MS mapping. We present the experimental collection of spectral and optical data from a murrina glass sample, and Greenlandic and Antarctic ice core samples, using LA-ICP-MS. An overview of data collection and use is shown in Fig. 1. Each dataset consists of spatially coherent optical and chemical maps. Application data are collected as sparse chemical maps, while validation data are collected as full chemical maps and are artificially masked to remove data.

We apply four inpainting approaches to approximate missing data in chemical maps. Two inpainting approaches, one channel Telea (1C Telea) and one channel neural network (1C NN), operate solely on the spectral data, and are referred to as one-channel approaches. Where available, additional data can be used to guide inpainting. To demonstrate this approach, two more inpainters are presented that use an additional optical data channel to give the inpainting algorithms additional context. These two-channel approaches, two channel mean replacement (2C MR) and two channel neural network (2C NN), exploit the harmony between optical and spectral data by conditioning the inpainting on a binary mask separating regions based on optical data. Two methods, 1C Telea and 2C MR, utilise traditional inpainting methods, while the other two, 1C NN and 2C NN, use a simple neural network architecture trained on an experimentally acquired dataset. While demonstrated on glass and ice core samples, these approaches can be extended to other matrices measured using LA-ICP-MS.

### 2.2 Experimental data collection

#### 2.2.1 Mapping procedure

**Fig. 2:**
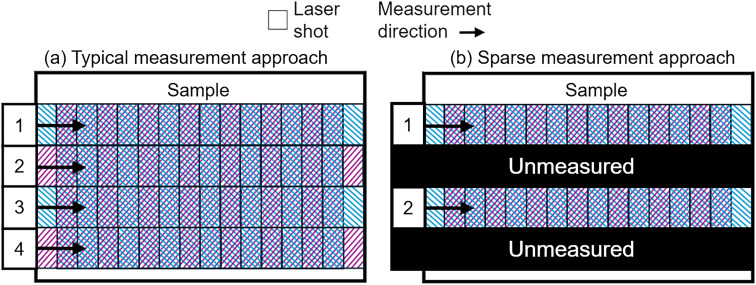
Typical LA-ICP-MS measurement pattern (a), and sparse measurement pattern (b). The overlap between adjacent spots is illustrative of the dosage, *n*, in this case with *n* = 2. Numbering indicates the order in which rows are measured. The pattern in (a) returns a chemical map with ID of 1, while the pattern in (b), with every other line unmeasured, returns a map with ID 0.5.

Typically LA-ICP-MS mapping experiments are carried out using uni-directional scanning,^25^ collecting data line-by-line in a pattern shown in Fig. 2(a). This involves rapidly firing a laser at the surface of a target sample, and transferring ablated material to an ICP-MS system where its elemental composition is measured. Parametrising the time taken to complete a LA-ICP-MS measurement, *T*_M_ (s), over a surface area, *A* (m^2^), gives the expression:1
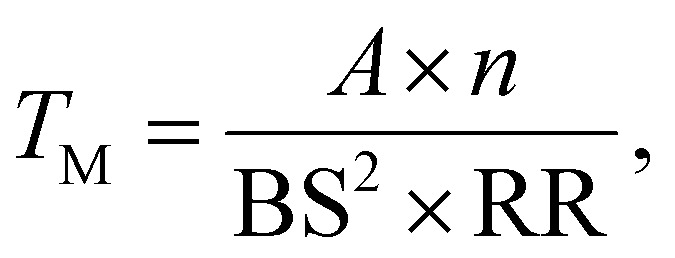
where *n* is the dosage (illustrated for *n* = 2 in Fig. 2), BS (m^2^) is the spot size of the laser, and RR (Hz) the laser's repetition rate.^25^ The return time of the stage to its initial starting location must also be considered. This establishes the total measurement time, *T*_T_, as:

2 *T*_T_ = *T*_M_ + *T*_R_,

where *T*_R_ is the total return time of the stage over the whole measurement. Assuming the distance between parallel points on adjacent profiles is much smaller than the distance between the start and end of the profiles, the total return time is dependent on the return time between a pair of adjacent profiles, *T*_r_, and the number, *N*, of profiles required to cover the entire target area:

3 *T*_R_ = *T*_r_(*N* − 1).

Using the expression for *T*_R_ from eqn (3) and *T*_M_ from eqn (1) in eqn (2) gives the expression:4
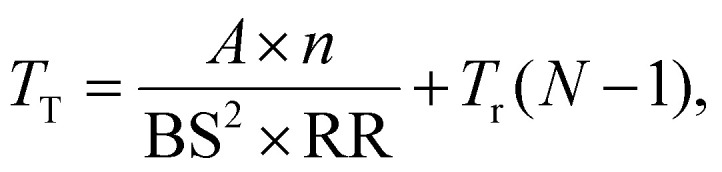
for the total measurement time for a map.

To reduce this measurement time, rows can be left out of the traditional sampling pattern, as illustrated in Fig. 2(b), reducing the information density (ID) of the measured area. An ID of 1 represents a fully mapped area, while an ID 0 represents a wholly unmeasured area. In the limit where the number of measured rows remains large (where 
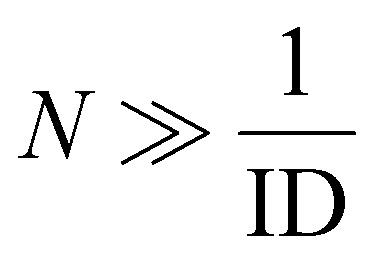
), the total measurement time can be approximated as:5
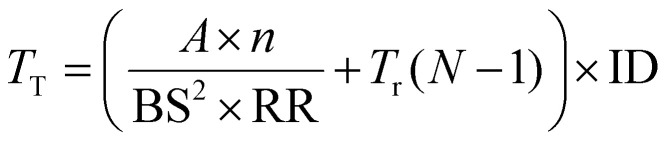
and tasks inpainting approaches to replace the unmeasured rows in the spectral data, restoring them to an ID of 1.

#### 2.2.2 Murrina glass data

A murrina sample was measured using a newly established LA-ICP-TOF-MS system at the Alfred Wegener Institute Helmholtz Centre for Polar and Marine Research, Bremerhaven, Germany (AWI).^26^ This setup utilises an *Iridia* 193 nm excimer laser ablation system, here using a long pulse module (Teledyne Photon Machines), coupled to a *Vitesse ICP-TOF-MS* (Nu Instruments) using an ARIS (Teledyne CETAC Photon Machines) for rapid aerosol transfer. While a large mass range is measured in the ICP-TOF-MS, only the *m*/*z* = 56 (corresponding to Fe) is focused on in this study as it displays variability over both large and small scales.

Glass data were collected on two separate days. Multiple 2D impurity maps, with full chemical maps with an ID of 1, and sparse maps with IDs of less than 1, of mm-sized areas on the sample's surface collected with a laser spot size of 10 μm. We used a firing rate of 500 Hz, dosage of 10, and a fluence of 4 J cm^−2^. Optical mosaics of each measured area were taken using the integrated optical camera, which is co-axial with the laser system.

#### 2.2.3 Ice core data

The majority of ice data utilised in this study are a compilation of measurements taken from different samples using the LA-ICP-MS system at the University of Venice (UNIVE). We utilise a total of 20 chemical (ID = 1) and optical data pairs collected between November 2022 and November 2023 on a total of 8 Antarctic and Greenlandic ice intervals. The samples originate from the EDC,^27^ EGRIP,^28^ and RECAP^29^ ice cores. Each measurement was carried out observing evolving best practice^19,30^ while following the same fundamental approach.

The system at UNIVE consists of an Analyte Excite ArF excimer 193 nm laser (Teledyne CETAC Photon Machines) connected *via* an ARIS rapid aerosol transfer line to an iCAP-RQ ICP-MS (Thermo Scientific). Samples were hosted in a HelEx II two-volume ablation chamber on a cryo-stage capable of maintaining the solid state of samples during analysis. This setup required samples to be prepared with approximately 1 cm thickness, with varying widths of 2 cm and below, and length not exceeding 9 cm.

Measured 2D impurity maps of square millimetre size were acquired with a spot size of 20 or 40 μm, firing rates of between 200 and 300 Hz, dosages of 9 or 10 (adjusted to synchronise material sampling with the ICP-MS measurement cycle), and fluences of between 3 and 4.5 J cm^−2^. As some spectral measurements on ice samples were collected with a 20 μm laser spot and others with a 40 μm laser spot, the higher resolution data were downsampled to 40 μm. Given its archetype as a soluble-in-ice elemental species we primarily focus on Na (*m*/*z* 23) in this study, although other elements were also recorded.

In contrast to most ice measurements being carried out using the UNIVE system, an exemplary application dataset was collected using the system at AWI, with a spot size of 40 μm. Cryo components were integrated into this system^26^ to ensure the sample remained frozen during measurement. This data set comprises a full chemical map, with adjacent regions collected with IDs of less than 1.

### 2.3 Data processing

Spectral data collected from the ICP-MS underwent standard drift and background corrections and subsequent exporting into 2D maps using the software HDIP (Teledyne CETAC Photon Machines). Intensities adjusted to negative values during background correction are subsequently assigned a value of zero. Chemical maps, resulting from combining the spectral data with spatial data from the LA system in HDIP, detail relative chemical intensities on the surface of the measured sample and remain uncalibrated.

The murrina sample has visible variability in the optical data allowing a binary mask separating adjacent regions to be hand-traced. Similarly, ice sample optical data show dark lines identified as GBs, which are hand-traced to produce a binary mask isolating the GB network from the grain interiors. These segmentations are downsampled to the same resolution as the chemcial maps, 10 μm for glass, and 40 μm for ice. Joining the chemistry channel and downsampled optical segmentation gives a combined two-channelled image.

### 2.4 Training, validation, and application dataset

To train the NNs a subset of one and two-channelled images with full chemical maps were isolated to serve as the ground truth data set. These training and validation data had regularly-spaced rows of the chemical maps artificially masked to provide an analogue to sparsely collected spectral data. The effect of obscuring rows in the maps is to reduce the ID in the map to between 0 and 1, analogue to experimental data collection. These masked maps are subsequently inpainted, and the inpainted output compared to the original unmasked data, providing a ground truth against which the inpainting performance is quantified. Obscuring and inpainting are carried out only on the chemical maps; optical data and derived masks always have an ID of 1, representing a suggested experimental procedure of full optical data collection and sparse chemical mapping.

## 3 Inpainting methods

### 3.1 Traditional inpainting

The first of four implemented inpainting methods, 1C Telea, utilises the well-established Telea algorithm^9^ to inpaint missing data based on the spectral channel only. The algorithm works iteratively from the outside of a missing region inwards by taking a weighted sum of pixels in the neighbourhood of the missing pixel, approximating its value.

The second applied inpainting method, 2C MR, is a custom-implemented approach based on the Telea algorithm that also incorporates optical data alongside spectral data. The added mask channel allows the classification of pixels into two distinct regions. In the case of the murrina samples, adjacent optically distinct regions are classified separately, while for ice samples regions are classified as either GB or grain interior. During inpainting, a target pixel is replaced by the mean of neighbourhood pixels with the same categorisation as itself. Unlike the 1C Telea approach, no iterative operation from outside the region inwards is implemented. To manage situations where no pixels of the same category are adjacent to a target pixel, the neighbourhood size increases with decreasing ID.

### 3.2 Neural network inpainting

**Fig. 3:**
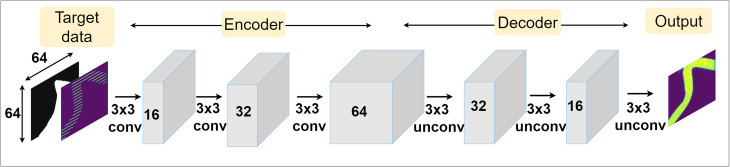
Design of the NNs used to inpaint missing regions in chemical maps. The network operates on input patches of size 64 by 64, with the 2C NN approach taking both the chemical map and optical mask channels as input, while 1C NN takes only the chemical channel. The network uses an encoder and decoder to increase and then decrease the number of channels sequentially. Only the chemical channel is considered at the output. The non-masked input data is overlaid on the output before use.

The third and fourth inpainting approaches, 1C NN and 2C NN, each use a purpose-implemented NN for inpainting. In both cases, the NN utilises the same simple architecture illustrated in Fig. 3 with an encoder followed by a decoder, resembling a very simple context encoder or autoencoder^31,32^ with no reduction in patch size but changes in image dimension.

During application, data with ID of less than one, either experimentally acquired or artificially masked from full chemical maps, are inpainted in patches of 64 by 64 pixels. These patches are re-arranged to the original full maps to form the final output. To avoid artifacts at the edge of patches after reconstruction, patches are generated with small overlaps and reconstructed intensities in edge regions are averaged. Analogue to the processing applied to empirically collected data, the output of the NN has pixels with values less than zero set to zero. The NNs output an inpainted image with an ID of 1, with every row in the image approximated by the network. This image subsequently has the input data (ID < 1) overlaid to form the final output of the NN approaches, a hybrid of the measured and approximated rows.

Two NNs, one for 1C NN and one for 2C NN, are separately trained a train in data set, with four networks trained in total, two on glass reference data and two on ice reference data. This learning uses supervised training. To produce a training dataset, chemical maps were masked to generate data with IDs between 0.2, representing the case where every 5th row is measured, and 0.8, representing data where every 5th row is omitted from measurement. The masked chemical maps and their corresponding optical masks are split into 64 by 64-pixel square patches and fed in batches of 32 as reference examples to the NNs during training. These patches cover the entire measured area and have some overlap to allow a larger training set to be produced.

To quantify a network's performance, the difference between the output chemical map generated by the NN and the ground truth chemical map is considered. The difference between the images is calculated as their pixel-wise mean squared error (MSE) across the whole image area, which is used as the reconstruction loss function the network attempts to minimise. Network weights are iteratively adjusted based on this loss, and, therefore, the network learns to minimise the pixel-by-pixel variance between the ground truth patches and its output patches.

### 3.3 Validation and application

To determine the suitability of each implemented inpainting method their performance must be assessed. This validation uses data previously unseen by the NN approaches during their training. The performance of each approach is quantified by considering the MSE between the image output from each inpainting technique and the ground truth data. The MSE is calculated only in the inpainted areas and gives a simple pixel-by-pixel quantification of variation between the two images but does not capture structural information. Visual inspection of images is also used to assess inpainting performance. The small and large-scale variations captured by each inpainting approach can be observed and discussed, giving a further view of the operation and suitability of each approach.

To demonstrate an application of inpainting, experimental datasets comprised of full optical data and sparse chemical maps are collected. These chemical maps are then inpainted using all four described inpainting methods. Given the nature of these data as true applications of the method, there is no ground truth chemical map to assess their performance against.

## 4 Results

### 4.1 Data collection and processing

**Fig. 4:**
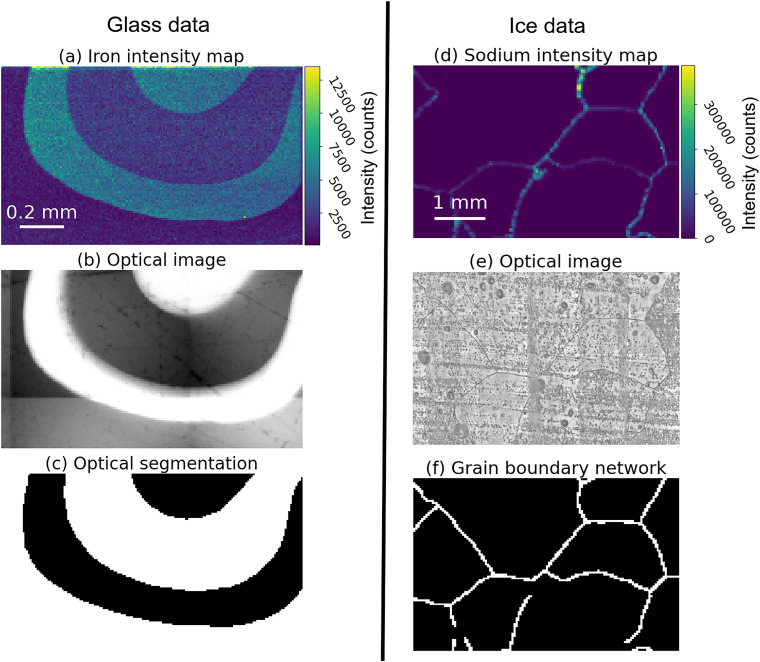
Murrina glass (first column) and ice (second column) validation data, showing the full chemical ((a) and (d)) and optical data ((b) and (e)), with masks resulting from hand-segmenting the optical data ((c) and (f)). White areas in (c) separate adjacent black areas of variable brightness in (b), while (f) represent where GBs are seen in (e). The ice dataset was collected from EDC ice from approximately 1096.7 m depth, with a small subset of the full measured area shown for readability.

The validation dataset for the murrina sample is shown in Fig. 4. This data shows typical elemental variability expected from a murrina sample, with distinct elemental intensity changes between regions. The optical data (Fig. 4b) was suitable for tracing segmentation masks, such as that in Fig. 4(c), which separates adjacent areas of light and dark colour. The NN training dataset for murrina samples consists of one chemical map, which was collected during the same measurement session as the application data.

For ice core samples, the dataset isolated for validation is a full optical image and chemical map collected from an EDC ice sample from 1096.7 m depth, and is shown in Fig. 4. All ice sample data had grain boundary masks, such as that shown in Fig. 4(f), traced by hand using a digital tablet and image editing software to produce a binary mask, taking approximately 1 minute for a square millimetre. The ice application dataset was collected from an ice sample which originated from a depth of 1096.7 m in the EDC core.

### 4.2 Network training

Each network was trained for 60 epochs, with batches containing 32 example patches from the target matrix. An initial learning rate of 0.001 was used for the 1C NN networks and of 0.002 for 2C NN; this rate was halved after every 20 epochs. The resulting change in MSE loss for each epoch for all four trained networks are plotted in the ESI.† The epoch used for the application was determined by inspecting the training plots. For glass, this inspection led to epoch 20 for both the 1C NN and 2C NN. For ice epoch 23 was chosen for the 1C NN, and epoch 40 for the 2C NN.

### 4.3 Performance validation

**Fig. 5:**
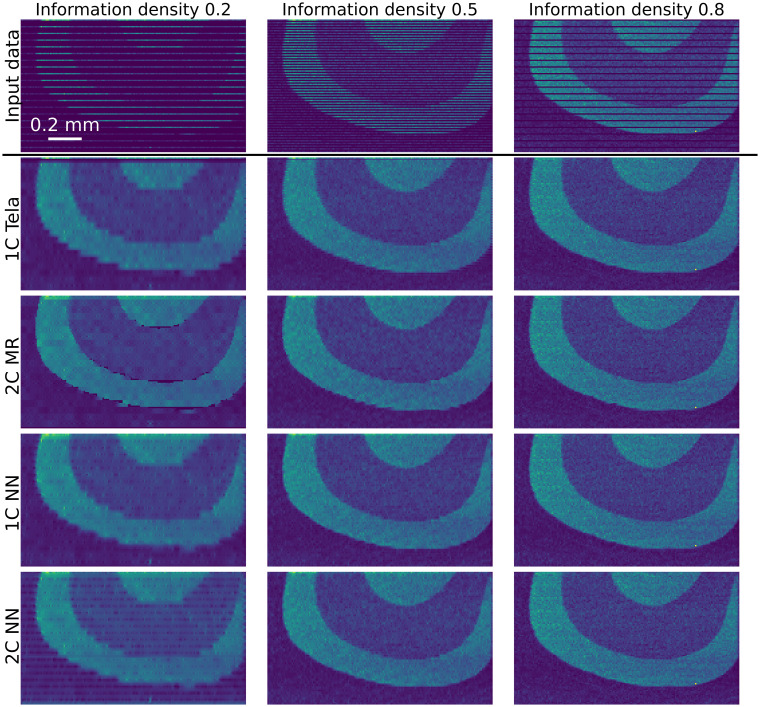
Illustration of the validation of inpainting performance on chemical data collected from a murrina glass sample. The masked chemical data in the first row originates from the full map shown in 4(a). Subsequent rows show the inpainted outputs for each of the four implemented inpainting algorithms, applied to the input data in the same column. The scale bar in the top left applies to all panels.

Validation examples for the performance of the inpainting algorithms on the murrina sample are shown in Fig. 5. This data originates from the ground truth chemical map (Fig. 4(a)), with data masked to different IDs before testing, with example-masked data in the top row of Fig. 5. Applying the inpainting techniques to these masked data returns the results displayed in subsequent rows of Fig. 5, with each row showing the output of a different inpainting technique. Visually, comparing Fig. 4(a) and each panel in Fig. 5 indicates that higher IDs lead to inpainting outputs more similar to the input data. At high input IDs of 0.5 and 0.8, all approaches perform similarly well, with variation between regions captured and region boundaries well defined. At an input density of 0.2, differences in output appear. 1C Telea blurs region boundaries, while 2C MR has patches with poor quality reconstruction, possibly due to slight offsets in the chemical map and optical mask.

**Fig. 6:**
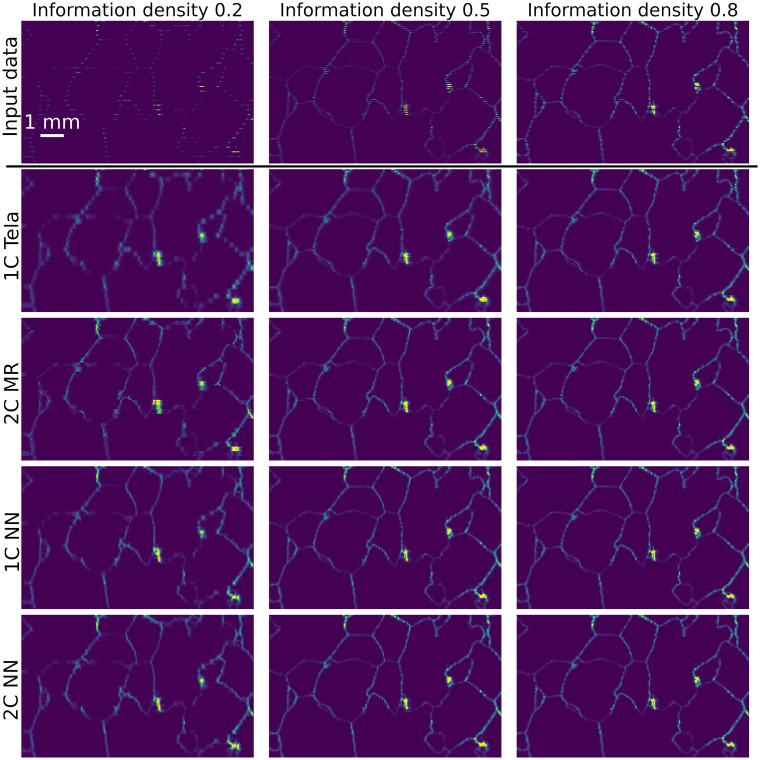
Illustration of the validation of inpainting performance on the ice core sample from the EDC core, depth 1096.7 m. The masked chemical data, taken from an expanded area of the plot shown in Fig. 4(d), for three different IDs is shown in the top row. Subsequent rows show the inpainted outputs for each of the four implemented inpainting algorithms. The scale bar in the top left applies to all panels.

Similarly, the inpainting techniques were applied to the validation ice data, a spatial subset of which is shown in Fig. 4(d), as illustrated in Fig. 6. There are visual differences between the outputs. Comparison between Fig. 4(d) and Fig. 6 shows the input and output are visually more similar for data inpainted with higher ID for all approaches. The large bright spots are good performance indicators, with less well-performing cases showing these regions smudged into their surroundings. The high intensity of grain boundaries bleeds into the grain interiors for the 1C Telea approach at all input densities. This effect is constrained by the added optical information provided to 2C MR. Both NN approaches produce visually smoother outputs than traditional approaches, with fewer missing intensity regions.

**Fig. 7:**
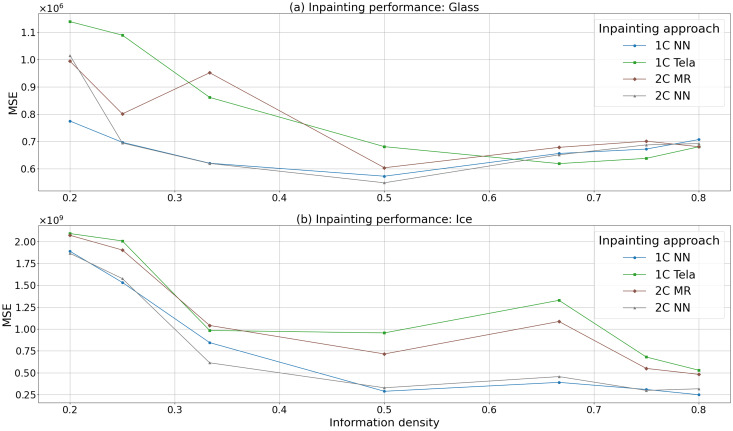
Plot of the performance validation of (a) glass and (b) ice for all inpainting techniques showing the MSE against input ID. The assessment is carried out by inpainting masked data generated from the chemcial maps in Fig. 4(a) for glass, and (d) for ice. Input data and inpainting outputs for IDs of 0.2, 0.5, and 0.8 are shown in Fig. 5 and 6. The difference in *y*-axis scale between the panels arises from different numbers of counts in measured maps arising from differences in sample properties.

For both glass and ice samples, the performance of each inpainting approach is quantified in Fig. 7 with the MSE between the inpainted regions and corresponding regions in the ground truth chemical map plotted for each approach. This plot contains the cases shown in Fig. 5 and 6, alongside results for other un-visualised IDs. The different absolute MSE values are not comparable between applications to glass and ice, as different elemental concentrations, and therefore instrumental intensities, are expected between the materials.

The general trend in Fig. 7 is that MSE values increase with decreasing ID, showing less faithful reconstructions of the ground truth. Small deviations from this trend are seen in cases where critical information is missing from obscured maps. When applied to glass, the traditional approaches perform the best above an ID of 0.6, below which the neural networks perform the best. The two channel approaches generally perfom similarly to their respective traditional/NN counterparts for all input IDs.

For ice, the neural network inpainters perform consistently well, with better performances than the traditional approaches across the entire range of information densities. 2C MR generally out performs its one channel counterpart, 1C Telea across all IDs while there is a similar performance for the neural network inpainters.

### 4.4 Application

**Fig. 8:**
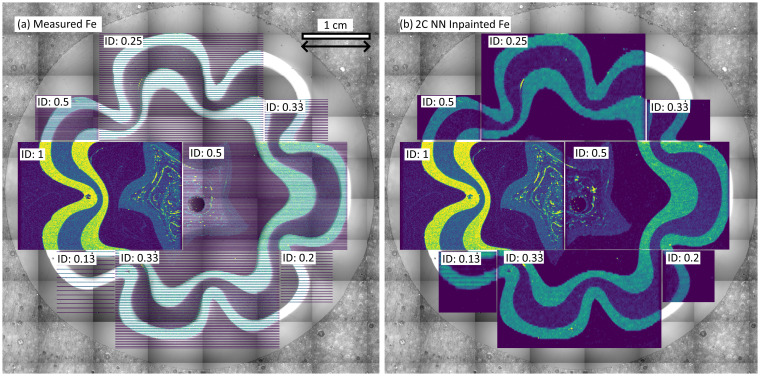
Chemical maps superimposed on top of optical data collected from the murrina sample. Spectral data is measured at a range of IDs (a), and the 2C NN used to restore the regions to approximated full images (b). Results for the 1C NN, 2C MR, and 1C Telea inpainting methods are contained in the ESI.† The ID for each rectangular region is indicated in the upper left of the region, the region collected with ID = 1 is shown for reference and is not inpainted. The spatial scale bar is applicable across both panels.

Spectral data were collected from both an ice sample and the murrina sample at a range of IDs to demonstrate the practical application of inpainting. For both target matrices, measurement time scaled linearly with information density. The results from measuring the murrina sample are shown in Fig. 8(a), with the inpainted output from the 2C NN shown in (b). Similar to the validation data, the decrease in performance with decreasing input ID is visible, with the data measured at an ID of 0.5 showing the most faithful reconstruction.

**Fig. 9:**
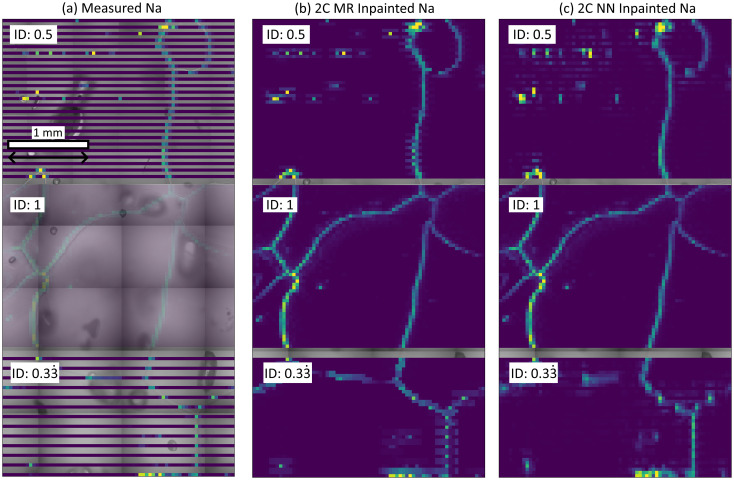
Chemical maps superimposed on top of optical data collected from an ice sample collected from EDC at a depth of 1096.7 m (a). Spectral data is measured at IDs of 1, 0.5, and 0.33 (a), and the 2C MR (b) and 2C NN (c) methods used to restore the regions to approximated full images. Results for the 1C NN and 1C Telea inpainting methods are contained in the ESI.† The ID for each rectangular region is indicated in the upper left of the region, the region collected with ID = 1 is shown for reference and is not inpainted. The chemical plot in the ID 1 region in (a) is transparent to allow some visibility of optical data behind. The spatial scale bar in the top left is applicable across the whole figure.

The example data for the ice sample is shown in Fig. 9, with sparse data shown in (a) and the output of the inpainting approach 2C MR shown in (b) and 2C NN in (c). The inpainting results from the 0.5 ID input is, visually, significantly better than for the 0.33 ID input, with boundary intensities much more continuously populated. The 2C MR approach appears to reproduce intensities along horizontal boundaries better than vertical boundaries, with 2C NN showing the opposite trend.

## 5 Discussion

### 5.1 Data collection

As illustrated in Fig. 4, which shows the chemical ((a), (d)) and optical ((b), (e)) channels, there is good spatial co-registration between the optical and spectral data, allowing effective masking of spectral data based on features in the optical images. The measured ice samples have a range of chemical and physical properties, including concentrations of measured Na and mean grain size, which aids the generalisation of the trained inpainting models. Conversely, only the fully mapped area in Fig. 8(a) is available as training data for the glass sample. Given the relative simplicity of features in the chemical maps measured on glass which are all captured in the training dataset, we are confident that this small dataset is large enough for basic training. However, a larger training data set collected from a range of similar glass samples could increase the performance of the implemented NNs and would be vital if such a network were to be applied to other glass samples with different chemical variability.

For both glass and ice samples, the chemical maps collected have approximately hundreds of pixels in each dimension, making patch sizes of 64 by 64 suitable for use in the NNs. The suggested data collection scheme of leaving out evenly spaced rows during measurements is easily implemented on both LA-ICP-MS systems used, with examples of the output of such experiments shown in the application datasets in Fig. 8 and 9(a).

### 5.2 Network architecture and learning

Within the range of available inpainting architectures,^33^ the choice of architecture related to an autoencoder^32^ or a context encoder is well motivated. Similar architectures to a context encoder, which is designed to repair patches in colour images, can be used to reconstruct arbitrary sizes and shapes of missing regions.^31^

Deeper learning architectures, including those using layers to reduce image size, u-net-like cross connections, and implementations of bottlenecks, were tested but resulted in worse network performance. This is likely due to the relatively small training dataset these models are trained on, and the small size of patches the network operates on. Small training datasets can lead to overfitting of complex NN architectures, leading to good performance on training data but bad generalisation to application data. As the training dataset is very small for the glass NNs the network will likely generalise worse than the ice NNs. As only one murrina with a relatively simple chemical distribution was measured for this study generalisation is less important than for the application to ice. In the case of ice, the networks were trained on data collected from a relatively large number of samples. This dataset can be added to in the future, furthering the generalised performance of such networks. Increasing training dataset size could allow more complex networks to be trained, for example those using skip connections in a u-net like architecture allow features to be preserved between network layers. Such architectures may also benefit from increasing the patch size to larger than 64 by 64 pixels to include more contextual information in each patch. The option to utilise more complex network architectures in the future motivates collection of larger datasets, with both an increased number of example maps and of the dimensions of each map.

The NNs’ training curves (shown in the ESI†) for the networks trained on ice data, show suitable training behaviour with an expected decrease in loss, followed by a convergence to stable performance. The flattening of the curves and reduction in oscillation after epoch 20 can be explained by the corresponding reduction in learning rate.

### 5.3 Inpainting performance validation

The performance validation for both glass and ice illustrated in Fig. 5 and 6 respectively, and quantified in Fig. 7, highlights the strengths and weaknesses of each approach.

All approaches show good performance for IDs above 0.5 on glass and 0.33 on ice, as quantified in Fig. 7. This suggests that in cases where only small amounts of data require inpainting, for example, where small regions are corrupted or otherwise missing, all approaches can be used. As both 1C Telea and 2C MR do not need a training dataset they can be applied to data immediately they are well positioned for such reconstructions. These approaches can be applied in cases where detector saturation or other instrumental factors (such as temporary loss of instrument connection, stage tripping, or lack of focus) lead to small missing areas of data, which can be quickly and reliably approximated using traditional inpainting approaches.

For IDs below 0.5, the NNs perform best on glass, while they perform consistently the best on ice across all information densities. This places these approaches well for experiments where data is deliberately under-sampled and subsequently reconstructed. Collecting data at an ID of 0.5 represents an immediate saving of 50% of the laser shots used to fully map an area, with very little extra post-processing burden or reduction in output quality. The nature of these approaches as interpolations must be considered when interpreting inpainted maps. At the micro-scale, these approaches can not precisely insert features in unmeasured regions but do provide suitable impressions of larger-scale variability.

Added optical information can improve the performance of inpainters, with the 2C MR approach generally performing better than 1C Telea on ice. Visual inspection of inpainted outputs shows that 2C MR is sensitive to offsets in registration between the mask and chemical map. These offsets are often unavoidable due to differences between the optical and chemical data. For example, the ice GB mask can be slightly spatially offset from the spectral data, and the chemical width of grain boundaries is not identical to their optical or physical width. Therefore, two-channel methods resilient to offsets between the spectral data and GB masks, such as 2C NN which does not show such rigidity, are practical.

Exploration of further performance metrics reveal similar trends. A metric that considers structural, intensity, and contrast differences between images is the structural similarity index (SSIM).^34^ Previously explored when optimising 2D maps collected using LA-ICP-MS,^35^ this metric varies between −1 and 1, where two images with a SSIM of near to 1 are similar. Plots equivalent to those in Fig. 7 but showing SSIM are contained in the ESI,† and reveal similar strong performance of the NN approaches on ice samples. This metric also places 2C MR consistently higher performing than 1C Telea on ice across all IDs when tested on the ice validation sample.

Where better performances, as quantified by either MSE or SSIM are required, or different matrices that do not train well with this network structure are analysed, other approaches to inpainting may prove suitable. For example, generative approaches can be used to provide an adversarial loss alongside the reconstruction loss^31^ while masked autoencoders^36^ and diffusion models^37^ are also used for similar tasks. Where possible, using larger training datasets, potentially utilising transfer learning to fine-tune pre-trained networks, and/or using larger patch sizes may also return better generalisation.

Where computational power is important, after initial training of the NN approaches, the time taken for all algorithms except the 2C MR to run at is comparably fast. On a standard laptop computer, the ice validation data shown in Fig. 6 takes on the order of 0.1 seconds to inpaint using the 1C Telea, 1C NN, and 2C MR approaches. At low IDs, the 2C MR takes a significantly longer time to run (on the order of 1 second) due to crudely implemented requirements to increase the neighbourhood size to find nearby classified pixels drastically. This time-based performance motivates the use of the NN approaches and 1C Telea. The speed of 2C MR could be improved by developing a new method to utilise a Telea-like algorithm, incorporating fast inpainting while considering the boundary and interior regions separately.

### 5.4 Inpainting application

The demonstrated inpainting approaches show promise for practical application to both relatively simple matrices such as murrina glass and to more complex matrices such as ice. In the case of glass analysis, Fig. 8 shows that measurement at an ID of 0.5 collects representative chemical data, including capturing small scale variations in chemical intensity. Very low IDs of 0.2 could be used to rapidly map large areas during initial analysis of such samples, to identify regions of interest for future full-mapping analysis.

The potential for enhanced performance for inpainting approaches using optical data demonstrates the utility of such for studies of soluble chemistry in ice core research. Thus, it should be exploited where possible. Since optical data are already used for localisation studies *via* LA-ICP-MS,^38^ implementing an approach to collecting higher-quality, and therefore easily machine-segmentable, optical images co-axial with LA-ICP-MS data is of interest. Indicative early experiments, briefly described in the ESI,† on higher-quality optical data which shows GB and grain interior regions at higher contrast a high likelihood that automatic segmentation can be applied to such data using a purpose-trained segmentation u-net.

In the case of ice, there is a demand to analyse centimetre-square ice samples to investigate the paleoclimate. Such analyses require the collection of representative chemical data from hundreds of metres of ice. Achieving such an objective requires industrial-style high-throughput rates of measurement of ice samples if LA-ICP-MS is to be applied. The results shown in Fig. 9 motivates a hybrid approach to such measurements, using full-area mapping for both localisation studies and provide a training dataset for inpainting NN, coupled with sparse data collection and subsequent inpainting to cover large areas. This approach can be useful for applications covering large scales where further data processing will take place, such as paleoclimate signal acquisition through combination of parallel profiles.^20^ Averaging inpainted areas may produce more accurate palaeoclimate reconstructions than non-inpainted data, but this process is yet to be verified.

**Fig. 10:**
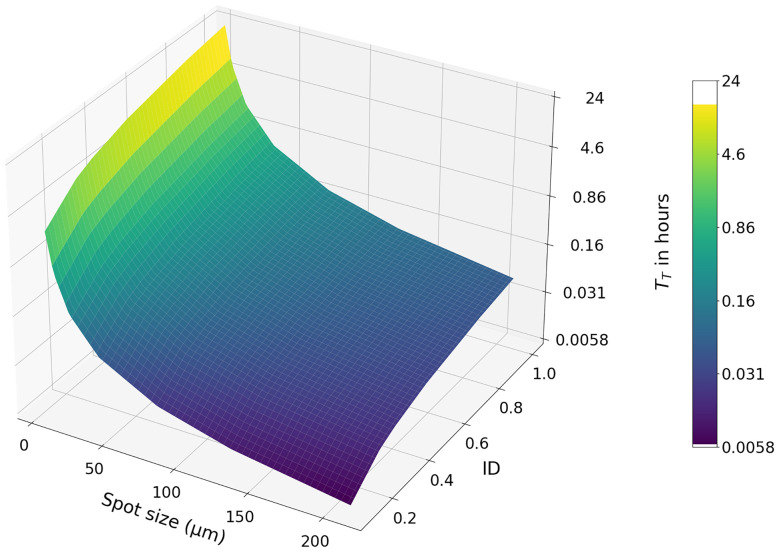
Visualisation of the time taken to measure a square-shaped area under different experimental conditions. Measurement time is calculated using eqn (5), with *A*, *n*, RR, and *T*_r_ set to constant values of, 1 cm^2^, 10, 500 Hz, and 3.5 s, respectively. ID and BS are varied, and *N* is set to the number of profiles required to cover the area based on the spot size. Note the time axis is on a logarithmic scale, with the colour map and *z* axis representing the same information.

An example case to demonstrate how *T*_T_ in eqn (5) changes based on variations in spot size and ID can be established by considering measurements over a square-shaped surface of 1 cm^2^. For a typical uni-directional area scan, *T*_r_ can be estimated by setting up measurements in the software used to design laser ablation scans and recording the projected measurement time. For the setup at AWI the software gives a *T*_r_ of approximately 3.5 s for each profile in this target geometry. Considering a typical dosage of *n* = 10 and a repetition rate of 500 Hz, eqn (5) can be used to calculate *T*_T_ under different experimental conditions, the results of which are shown in Fig. 10.

The surface plotted in Fig. 10 can be used to identify the spot size and ID required to achieve a target analysis time. Exploring such a parameter space allows an optimisation between measurement time, resolution, and ID to be determined. However, the plotted parameter space represents a simplification of the relationships established in eqn (5). This equation can be further explored to understand how measurement times can be reduced. For example, RR is often changed based on BS, while *T*_r_ is dependent on the geometry of the measured area and can be considered negligible in the case of a bi-directional raster scan. Experiments are best designed while considering this entire parameter space, and the addition of ID as a parameter is intended to add a further consideration to experimental design decisions.

### 5.5 Broader application

In theory, these inpainting approaches can be applied to other LA-ICP-MS target samples to facilitate large-area measurement or reduce time and resource consumption. To maximise inpainting performance, each target matrix will require careful design of experimental data collection and selection of inpainting method.

Interpolation approaches such as image inpainting are applied across scale and dimension. Inpainting is not restricted to the choice of spot size discussed in this analysis, with 10 μm chosen for glass and 40 μm for ice samples to match the specific application of LA-ICP-MS to these samples. These spatial scales can be adjusted based on the specific target application and matrix. Furthermore, given that the 3D manifestation of chemistry is important to understanding material composition and behaviour, such approaches could also be extended to facilitate 3D analysis. LA-ICP-MS has already proved a suitable method for collecting planes of spectral data from a 3D object.^39^ Gaps between adjacent layers collected during such analysis could be filled using similar inpainting techniques.

The range of analysed spectral channels can be extended and inpainting applied to calibrated data. The ESI† for this manuscript shows the results of training and applying the 2C NN inpainter to the *m*/*z* = 146 (Nd) and *m*/*z* = 23 (Na) channels measured on the murrina glass sample. In such cases where multiple chemistry channels are analysed, NN architecture could be adjusted to simultaneously inpaint all spectral channels simultaneously, with the extra information potentially aiding inpainting performance. Dimensionality reduction on groups of ICP-MS mass channel outputs could allow discussions of multiple channels at once. In the case of ice core analysis, a representative *boundary localised* channel could be isolated and discussed, rather than discussing channels individually. Although elements dispersed away from grain boundaries in ice samples shows less obvious spatial distributions, similar discussions are likely extendable to consider these components.

General image analysis toolsets can be shared and enhanced by considering different matrices and analytical techniques. Studies on biological samples, which can share similar structures to ice, are analysed using dedicated image analysis approaches which can be applied to other matrices.^40^ Furthermore, the chemistry of samples is often closely linked to patterns in other measured data, with optical and backscattered electron images having been used as reference points for LA-ICP-MS.^38,41^ Correlations between these data types can be exploited to propagate information about the chemistry into unmeasured regions, similarly to how the two channel inpainting approaches presented in this study utilise optical data to guide their inpainting.

## 6 Conclusions

Carefully designed experiments joined with computational post-processing can be utilised to save time and resources during LA-ICP-MS data acquisition. Experiments can be designed to collect only a fraction of the full spatial spectral data usually measured with LA-ICP-MS and inpainting techniques utilised to fill in the gaps, with often minimal difference in output. Such approaches can also benefit from harmonies with other more easily measurable data types, such as optical data, to guide approximations of spectral data. This approach to sparse data collection and subsequent inpainting is demonstrated on both glass and ice samples. These developments, coupled with the increased speed of LA-ICP-MS analyses, enable measurement of the large areas and high sample numbers required to fully collect high-resolution climate signals from highly thinned ice core samples. The presented approach is widely applicable to facilitate rapid data collection on a range of target matrices.

## Conflicts of interest

There are no conflicts of interest to declare.

## Supplementary Material

AN-150-D5AN00325C-s001

## Data Availability

Data availability statement for the article submitted to Analyst: For initial submission and during review, the underlying datasets have been made privately available to the editors and reviewers. These datasets contain the optical and chemical data discussed in the manuscript. The datasets will be made publicly available *via* open-access repositories such as those facilitated by, Pangaea (https://www.pangaea.de) or Zenodo (https://www.zenodo.org) after acceptance of the manuscript.
