# Supplementary material for: Faster chemical mapping assisted by computer vision: insights from glass and ice core samples
Source: Analyst. 2025 Jun 19;150(15):3408–22. doi: 10.1039/d5an00325c (PMC12198724; doi:10.1039/d5an00325c)
Supplement: AN-150-D5AN00325C-s001 [file AN-150-D5AN00325C-s001.pdf]

Supplementary Information (SI) for Analyst.
This journal is © The Royal Society of Chemistry 2025

S1 Extra inpainting results

Murrina inpainting

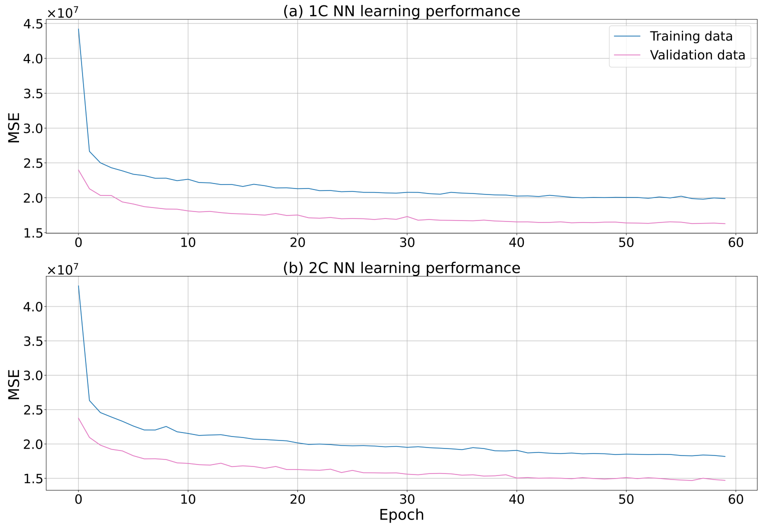

Figure S1: The average MSE loss between ground truth and output patches during training on murrina glass data, plotted per epoch for the (a) 1C NN inpainter and (b) 2C NN inpainter trained on the Fe channel. The legend indicating training performance on both training and validation datasets applies to both panels.

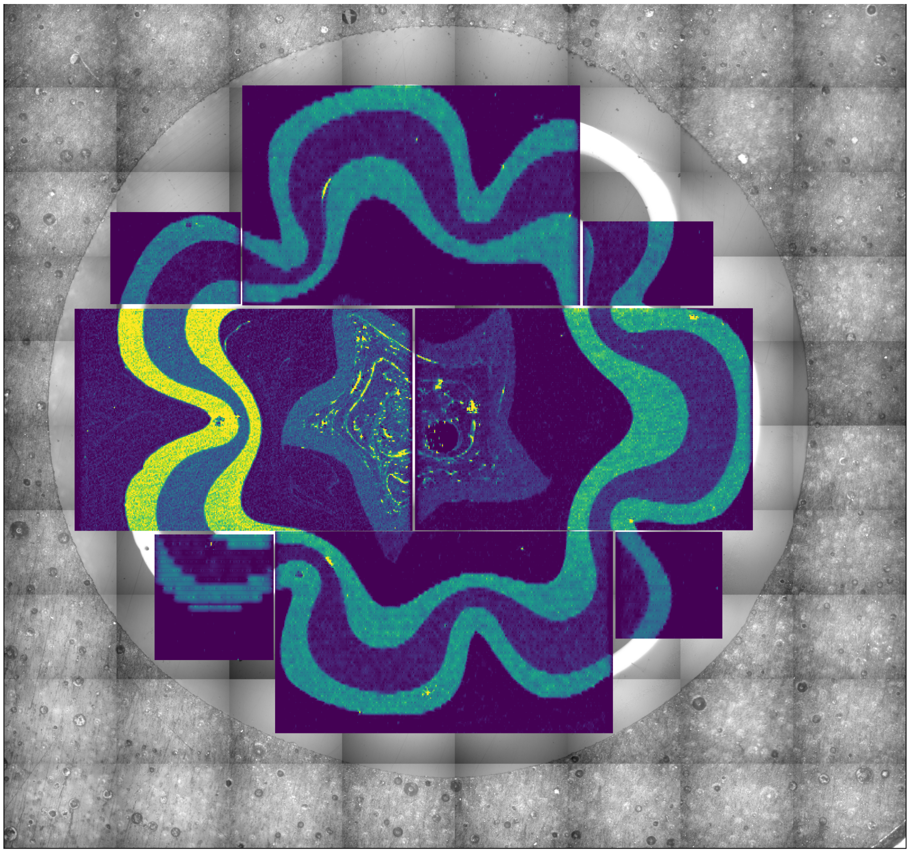

Figure S2: Inpainting results from the application of 1NN to the Fe dataset shown in figure 8.

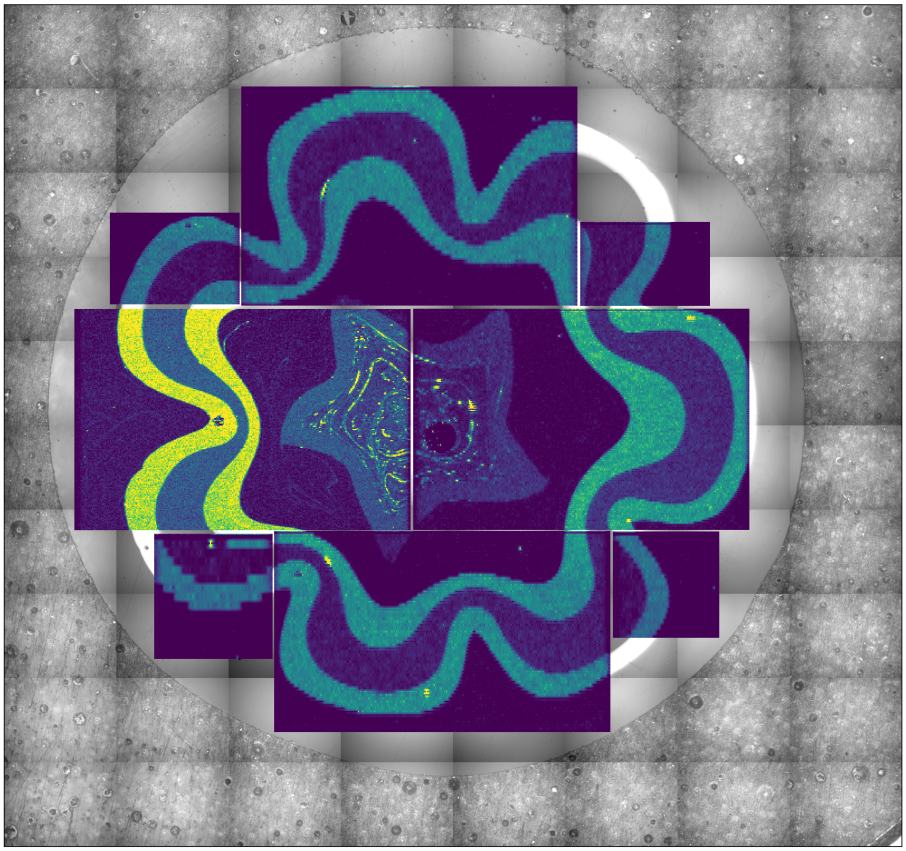

Figure S3: Inpainting results from the application of 1CV to the Fe dataset shown in figure 8.

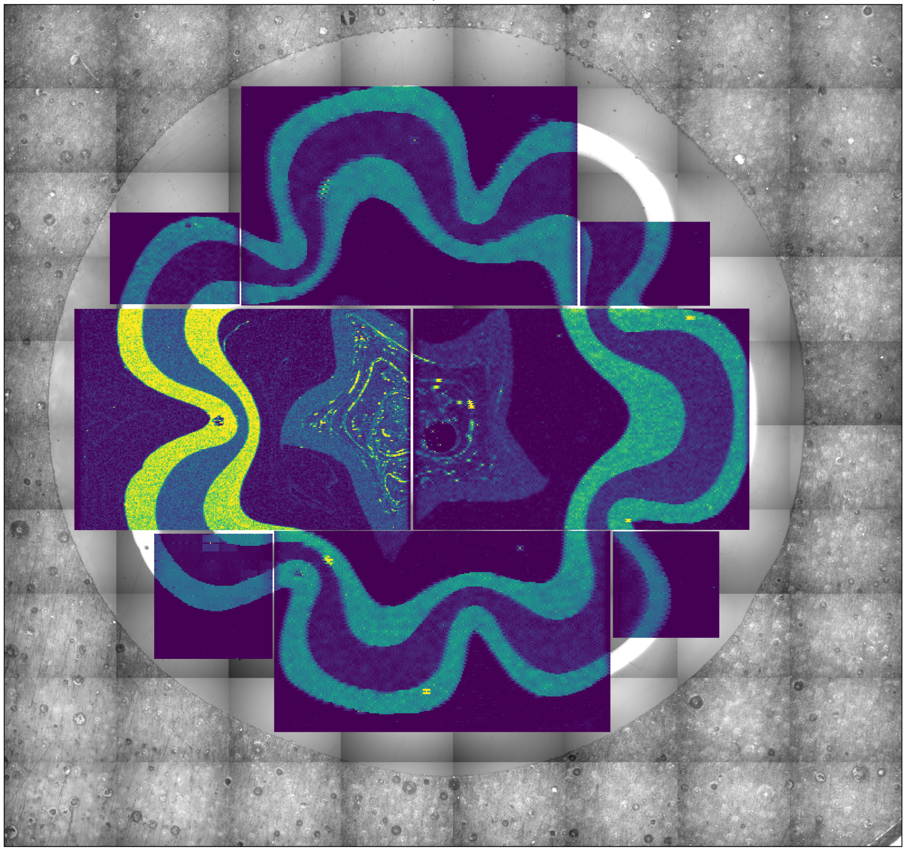

Figure S4: Inpainting results from the application of 2CV to the Fe dataset shown in figure 8.

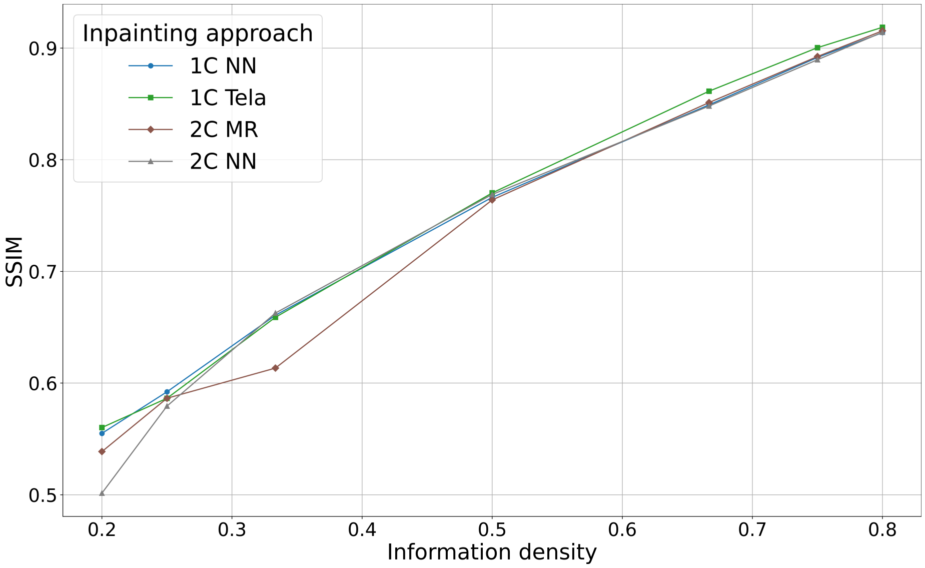

Figure S5: SSIM variation with input ID for Fe glass data, equivalent to Fig. 7 (a).

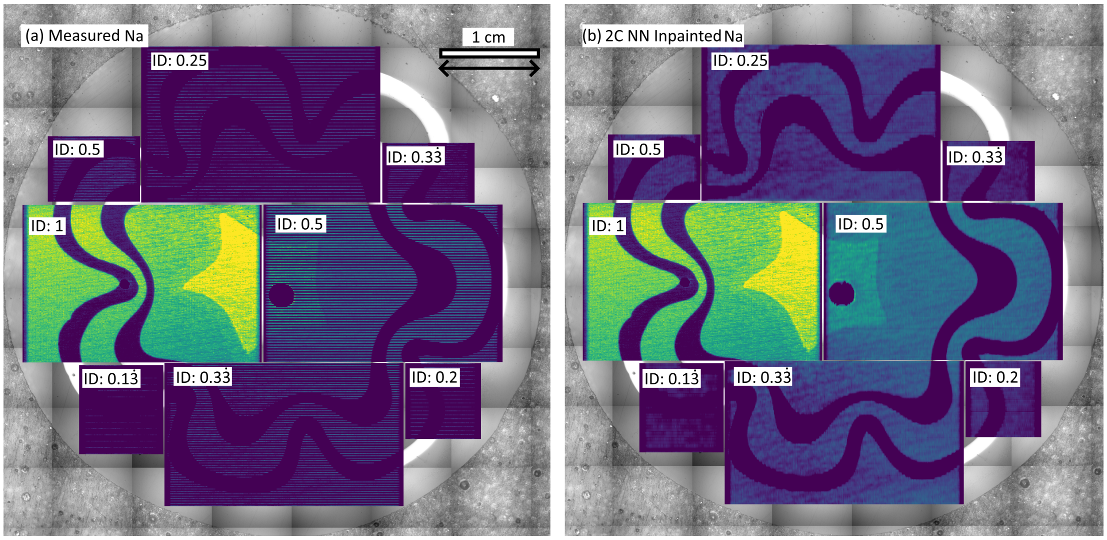

Figure S6: Inpainting results equivalent to figure 8 showing results from applying a purpose trained 2C NN to the m/z 23 (Na) channel.

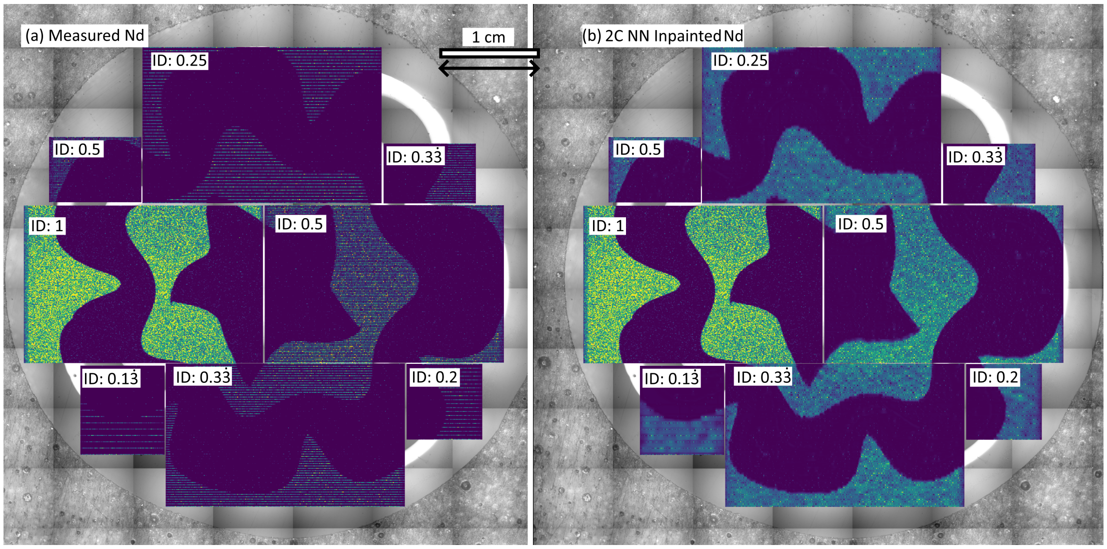

Figure S7: Inpainting results equivalent to figure 8 showing results from applying a purpose trained 2C NN to the m/z 146 (Nd) channel.

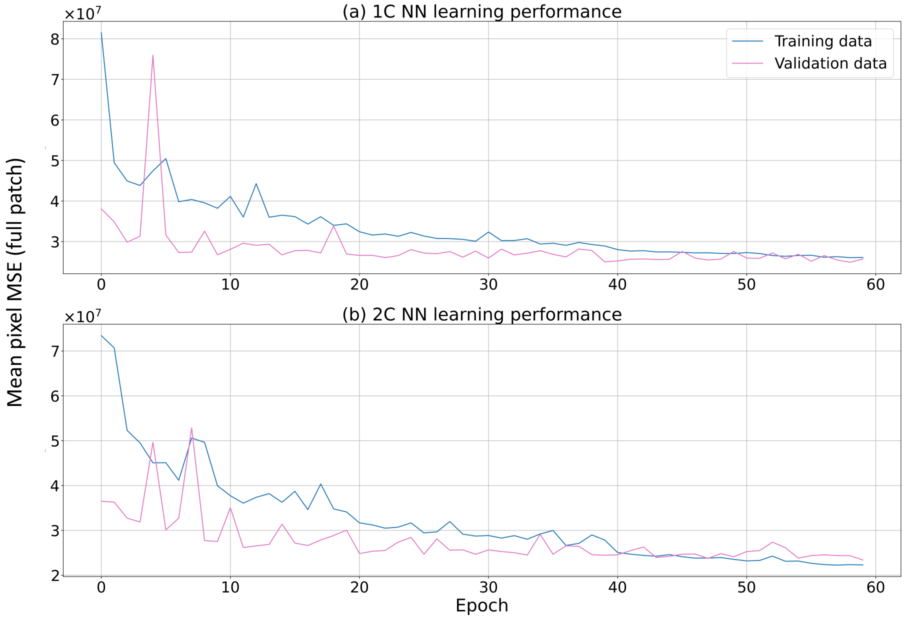

Figure S8: The average MSE loss between ground truth and output patches during training on ice data, plotted per epoch for the (a) 1NN inpainter and (b) 2NN inpainter. The legend indicating training performance on both training and validation datasets applies to both panels.

Ice inpainting

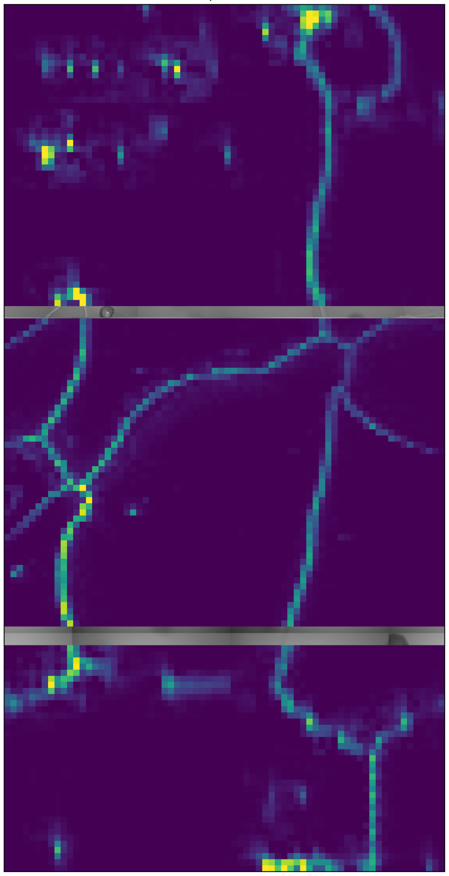

Figure S9: Inpainting results from the application of 1NN to the dataset shown in figure 9.

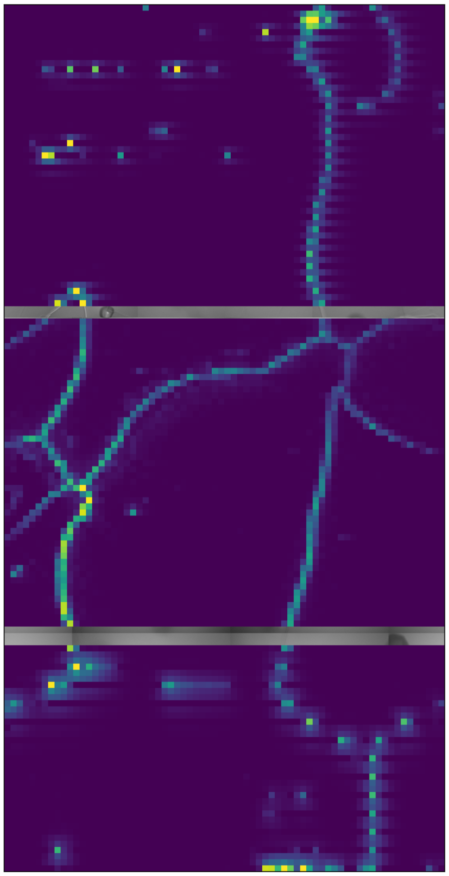

Figure S10: Inpainting results from the application of 1CV to the dataset shown in figure 9.

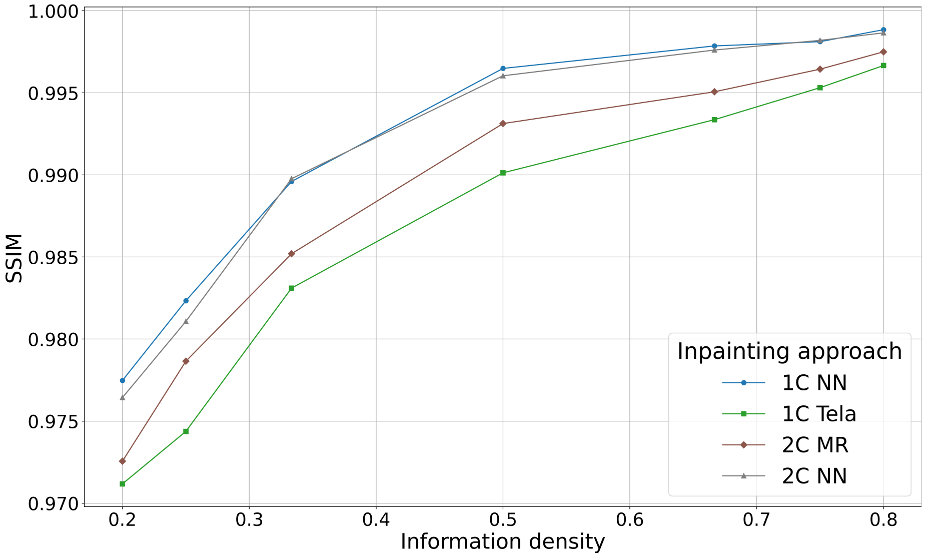

Figure S11: SSIM variation with input ID for glass data, equivalent to Fig. 7 (b).

S2 Optical data segmentation

A U-net segmenter, designed to segment out the grain boundary network from optical images taken of ice samples in a LA-ICP-MS system, has been explored. An indicative result of this early-stage development is shown in figure S12

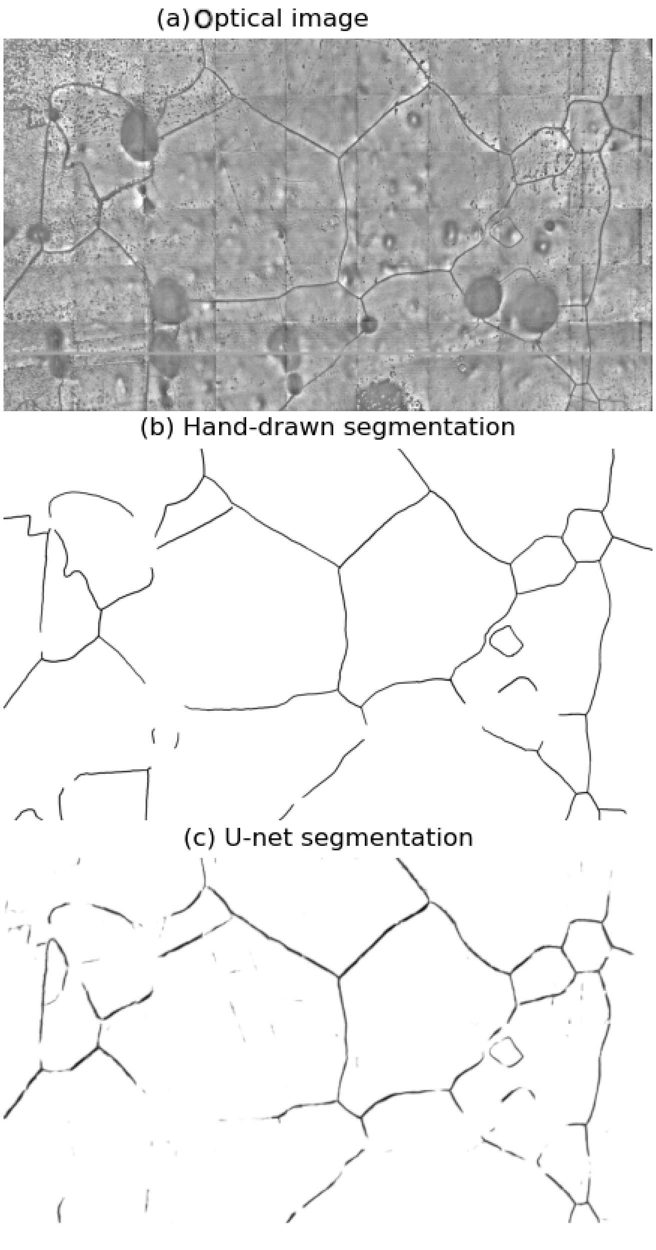

Figure S12: An optical image of an ice sample taken in a LA-ICP-MS system (a), a hand-drawn segmentation (b), and the output of a u-net intended to segment the grain boundary network (c).
